# Comparative secretome analysis of four isogenic *Bacillus clausii* probiotic strains

**DOI:** 10.1186/1477-5956-11-28

**Published:** 2013-07-01

**Authors:** Rosa Lippolis, Rosa Anna Siciliano, Maria Fiorella Mazzeo, Anna Abbrescia, Antonio Gnoni, Anna Maria Sardanelli, Sergio Papa

**Affiliations:** 1 Institute of Biomembranes and Bioenergetics, Italian National Research Council (CNR), Via Amendola 165/A, Bari, Italy; 2 Institute of Food Sciences, Italian National Research Council (CNR), Via Roma, 64, 83100, Avellino, Italy; 3 Department of Basic Medical Sciences, Neurosciences and Sense Organs, University of Bari, Policlinico, Piazza G. Cesare, 70124, Bari, Italy

**Keywords:** *Bacillus clausii*, Probiotics, Secretome, Proteomics, Two-dimensional Gel Electrophoresis, Mass Spectrometry

## Abstract

**Background:**

The spore-bearing alkaliphilic *Bacillus* species constitute a large, heterogeneous group of microorganisms, important for their ability to produce enzymes, antibodies and metabolites of potential medical use. Some *Bacillus* species are currently being used for manufacturing probiotic products consisting of bacterial spores, exhibiting specific features (colonization, immune-stimulation and antimicrobial activity) that can account for their claimed probiotic properties. In the present work a comparative proteomic study was performed aimed at characterizing the secretome of four closely related isogenic O/C, SIN, N/R and T *B. clausii* strains, already marketed in a pharmaceutical mixture as probiotics.

**Results:**

Proteomic analyses revealed a high degree of concordance among the four secretomes, although some proteins exhibited considerable variations in their expression level in the four strains. Among these, some proteins with documented activity in the interaction with host cells were identified, such as the glycolytic enzyme enolase, with a putative plasminogen-binding activity, GroEL, a molecular chaperone shown to be able to bind to mucin, and flagellin protein, a structural flagella protein and a putative immunomodulation agent.

**Conclusion:**

This study shows, for the first time, differences in the secretome of the OC, SIN, NR and T *B. clausii* strains. These differences indicate that specific secretome features characterize each of the four strains despite their genotypic similarity. This could confer to the *B. clausii* strains specific probiotic functions associated with the differentially expressed proteins and indicate that they can cooperate as probiotics as the secretome components of each strain could contribute to the overall activity of a mixed probiotic preparation.

## Background

*Bacillus* species (*B. cereus, B. clausii, B. pumilus*) are a large, heterogeneous group of Gram-positive, nonpathogenic, spore-forming microorganisms, used in many chemical, medical, and industrial processes taking advantage of their wide range of physiological characteristics and ability to secrete large amounts of extracellular proteins as well as biochemicals, antibiotics and other metabolites.

Mixtures of viable *Bacillus* spores have been marketed as probiotic preparations [1]. Probiotics are defined as microbial cell preparations or components of microbial cells that can beneficially impact human health. Probiotics have been shown to be useful in oral bacteriotherapy and bacterioprophylaxis of gastrointestinal disorders [2]. The positive effects of probiotics in human diseases can be associated with synthesis of anti-microbial substances, competition with pathogenic microorganisms, modification of toxins or toxin receptors, and immune system modulation [3].

The present proteomic study was performed on four O/C, SIN, NR and T *Bacillus* strains currently used as probiotics, which proved to be useful in treating various gastrointestinal disorders by improving the host intestinal microbial balance [4], and in preventing side effects in antibiotic therapy [5].

The use of the four *Bacillus* strains as probiotic species is supported by more than 40 years of clinical observations with excellent tolerability and no report of side effects. Moreover, *Bacillus* strains exhibit unique properties such as resistance to commonly used antibiotics [1], absence in normal resident intestinal flora and sporogenic activity [6,7]. *Bacillus* spores can survive in the acid gastric environment, and reach the intestinal tract where they germinate to vegetative forms [8,9]. Little is known on how these bacteria exert their therapeutic effects.

Recently, the four *Bacillus* strains have been characterized and catalogued as belonging to a unique genospecies identified as the alkali-tolerant species and aligned with members of *Bacillus clausii* subgroup, rather than with *Bacillus subtilis*, as previously reported. The four *B. clausii* strains display a low level of intra-species diversity and exhibit a high degree of genomic conservation [4], with inherent intrinsic difficulty in identifying the role of each strain in the probiotic function.

Our previous study described the proteomic profile of the four O/C, SIN, N/R, and T, *B clausii* strains and led to the recognition that the four strains, having the same genotypic traits, exhibited, surprisingly, variations in the expression level of several proteins [10]. This made possible to distinguish each of the four *B. clausii* strains based on their 2-DE protein signature.

In the present study, proteomics was applied to obtain a description of the extracellular proteome components (secretome) of the four probiotic O/C, SIN, N/R, and T *B. clausii* strains. Secreted proteins are involved in the hydrolysis of natural polymers [11,12], in processes like cell wall turnover, substrate binding or protein secretion [13,14] as well as in regulating the onset of post-exponential phase processes (competence, development and sporulation) [15,16]. As secreted proteins come into direct contact with host compartments, they can mediate host-bacteria interactions. Despite the potential importance of proteins secreted by probiotic strains, data on their identity are very limited.

Proteomic analysis and identification of secreted proteins differentially expressed in the four *B. clausii* strains could provide definite information on their functional features. The present study resulted in: i) detection and identification of differentially expressed secreted proteins, ii) identification of proteins related to specific probiotic functions associated with each strain, iii) a better understanding of how these microorganisms, characterized by a notable low level of intraspecies genome diversity, transfer their genetic information in the secretome expression profiles.

## Material and methods

### Bacterial strains

The four O/C, SIN, N/R and T *B. clausii* strains, now propagated for production of commercial probiotic preparation, were obtained from Sanofi Winthrop, (Milan, Italy) as separate spore suspensions. The designation of these strains was derived from their resistance to different antibiotic resistance markers: O/C was resistant to chloramphenicol, SIN was resistant to neomycin and streptomycin, N/R was resistant to novobiocin and rifampicin and T was resistant to tetracycline [1,17]. All strains were stored in a glycerol stock solution at -80°C.

### Strains and culture condition

Bacteria cells were plated directly from a glycerol stock solution onto the LB medium agar plate. (Tryptone 10.0g, Yeast extract 5.0 g, NaCl 5.0 g, H_2_Oad 1.0L) (Fluka, Buchs, Switzerland).

O/C, N/R, SIN and T *B. clausii* strains were maintained on LB-agar plates. The four strains were inoculated in LB medium supplemented with 100 mM tricine pH 8.0, from a stationary pre-culture. Growth was performed in 250 ml flasks containing 50 ml of broth at 37°C in an orbital shaker with radius of 5 cm at 150 rpm.

Bacteria growth was monitored by measuring the optical density at 595 nm. Cultivation was started with an initial optical density of about 0.04 at 37°C. Cells and culture medium (CM) were collected at early exponential growth phase (8 h), late exponential growth phase (16 h) and stationary growth phase (24 h) (Additional file 1: Figure S1).

The cultivation was performed in triplicate for each *B. clausii* strain. Cells from each independent triplicate were collected and proteins were extracted as described below.

### Validation procedures

Before proceeding with the proteomic analysis of secreted proteins, we verified that proteins detected in CM were not artifacts due to the presence of degradation products for spontaneous or inadvertent bacteria cell lysis or extracellular proteases degradation. To minimize degradation of secreted proteins by proteases, during CM collection and protein preparation, a protease inhibitor (1 mM phenylmethylsulfonyl fluoride (PMSF)), (Sigma-Aldrich, St. Louis, MO, USA) was included in CM.

To eliminate bacterial cells from CM, the supernatant was filtered through a 0.2mm low protein-binding miller filter (Millipore) before protein precipitation.

### G6PDH enzymatic assay

To control the possible release of intracellular proteins in CM, we measured the amount of the cytosolic marker glucose 6-phospate dehydrogenase (G6PDH) in CM and in whole cell lysate (WCL) by enzyme activity assay.

Bacterial pellets from a 50 ml culture, at stationary growth phase, were lysed by sonication in an ice bath for 10 × 30 s with a 30 s interval between each ultrasonic cycle, in 1 ml LB medium supplemented with the appropriate concentrations (1:100, [w/v]) of protease inhibitors cocktail (Sigma-Aldrich, St. Louis, MO, USA) to generate WCL. After clearance by centrifugation, serial twofold dilutions of the lysate were made in LB medium supplemented as above to yield final concentrations of 50%, 25%, 10%, 5%, and 1% (v/v).

The enzymatic activity of G6PDH in 100 μl of CM and in serial dilutions of WCL was measured spectrophotometrically at 30°C by following the rate of NADP^+^ reduction at 340 nm according to the method described by Bergmeyer *et al*. [18].

### SDS PAGE analysis of intracellular and secreted proteins

*B. clausii* OC, SIN, NR and T cells suspended in CM were collected from parallel sample preparation at stationary growth phase (24 h). After separating cells from CM, WCL was generated by sonicating the cell pellets. Briefly, cell pellets were solubilized in Laemmli buffer [19] without sodium dodecyl sulfate (SDS), bromophenol blue, and β-mercaptoethanol, supplemented with 1/100, (v/v) protease inhibitors cocktail. The suspension was sonicated as above. The lysate was cleared from insoluble material by centrifugation for 20 min at 12000 × rpm at 4°C. The resulting WCL supernatants and CM samples were precipitated with trichloroacetic acid (TCA) at 10% final concentration (w/v). Precipitates were solubilized in Laemmli sample buffer [19] and equal amount of proteins of CM and WCL were fractionated by polyacrylamide gel electrophoresis (SDS-PAGE) 12,5%.

### Intracellular and secreted protein sample preparation for 2-DE analysis

Samples of the four O/C, SIN, N/R and T *B. clausii* strains suspended the LB medium, were taken at the early exponential growth phase (8 h), late exponential growth phase (16 h) and stationary growth phase (24 h) (Additional file 1: Figure S1). Cell proteins were prepared as previously described [20], with some modifications. Briefly, cells were collected by centrifugation at 6500 rpm for 30 min at 4°C (Sorvall RT 6000 B, DuPont), from 50 ml of culture suspension. Cell pellets were washed twice with phosphate-buffer, suspended in lysis buffer (7 M urea, 2 M thiourea, 4% (w/v) CHAPS, 50 mM DTT, 0.5% (w/v), IPG-buffer (GE Healthcare) pH 3–10 supplemented with 1 mM PMSF and disrupted by sonication as previously described. Insoluble material was separated by centrifugation at 13000 rpm for 30 min at 4°C. Raw protein extracts were precipitated with three volumes of cold acetone, washed twice with cold acetone, air-dried and stored at - 80°C until use.

In order to obtain the secreted proteins, supernatants were subject to filtration through a 0.2 mm nitro-cellulose filter (Millipore, Billerica, MA, USA) and treated with 10% (w/v) TCA for 30 min. The aggregated proteins were precipitated by centrifugation, washed three times with 10 ml of cold 90% acetone, air-dried and stored at -80°C until use. Cell protein extracts and precipitated proteins from CM were dissolved in the lysis buffer and centrifuged (45 min × 15000 × g, 4°C) to remove any indissoluble material before two-dimensional electrophoresis (2-DE) analyses.

Protein concentration was determined using the Bio-Rad Protein Assay kit (Bio-Rad Laboratories, Hercules, CA, USA), according to the manufacturer’s instruction [21], with bovine serum albumin, as standard protein.

### 2-DE analyses

*B. clausii* intracellular proteins and CM proteins were separated by 2-DE essentially as described in Gorg *et al*. [22] and Hochstrasser *et al.*[23]. Isoelectric focusing (IEF) was carried out at 20°C with the Ettan IPGphor Isoelectric Focusing System (GE Healthcare) by using, in the first experiment, 24 cm immobilized pH gradient strips (IPG) with a linear pH gradient 3–10 (GE Healthcare) for an overview of total protein distribution pattern. To better resolve protein spots, in a second experiment, we used a non-linear (NL) 3–10 pH gradient strip. To zoom the specific region of the gel, IPG strips pH 4–7 were also used.

The IPG strips were rehydrated overnight at room temperature in a rehydration solution and 250 μg of protein sample was applied by cup loading. Focusing was carried at 90 kVh total. After focusing, the IPG strips were equilibrated for 15 min in the equilibration buffer (50 mM Tris/HCl, pH 8.8, 6 M urea, 30% glycerol, 2% SDS, trace of bromophenol blue) containing 1% DTT, and for further 15 min in the same equilibration buffer containing 2.5% iodoacetamide and 0.5% bromophenol blue. The second-dimension gel electrophoresis (SDS-PAGE) was carried out using the vertical slab separation unit Ettan Dalt II System (GE Healthcare). Homogeneous SDS 12.5% polyacrylamide gel was used in a Laemmli system [19] at a constant current of 15 mA gel^-1^ and at 10°C until the bromophenol blue dye front reached the bottom of the gel. Molecular mass markers and pI standards were from Bio-Rad. After separation, 2-DE gels were stained using Coomassie Blue Colloidal dye (Sigma-Aldrich, St. Louis, MO, USA), allowing quantitative comparison of spot intensit**y**. Bacterial cells were cultured in triplicate. CM from each biological repeat, i.e. resulting from three independent cultures, were collected before protein extraction and each protein sample was run in triplicate. (Additional file 2: Figure S2).

The eventual presence of proteinaceous compounds in the LB medium was excluded by 2-DE analysis of the LB protein component precipitated by treatment with 10% TCA (Additional file 3: Figure S3).

### Image analysis

Stained gels were scanned with an Image Scanner (GE Healthcare) at 300 dpi resolution to acquire the gel images that were analyzed with Image-Master 2D Platinum v.6 software (GE Healthcare). Spot detection was carried out using the optimized setting values for spot intensity, spot area and saliency determined by applying real-time filters in order to minimize the detection of artifacts and to maximize the real spot detection. After spot detection, manual spot editing was carried out to remove eventual artifacts that escaped the filtering process.

Three master gels from each O/C, SIN, N/R and T samples were used to create the four match.

Reproducible landmarks were used to match spots. Relative spot volume (% vol.), i.e. digitized staining intensity integrated over the area of the individual spot divided by the sum of volume of all spots in the gel and multiplied by 100, was used for spot quantification [24]. At the final level of matching, the four groups of gels were matched and a composite synthetic gel was obtained containing all spots present in at least two gels. The match identification number (ID) was used to identify all spots in a match. Spots present in all the gel images of one of the four classes but absent in the others, were considered to contain proteins expressed uniquely by one strain. Spots present in all the gels of the four classes and exhibiting an intensity difference between the four strains with a P value < 0.05, using the two-tailored Student’s t-test for equal or unequal variance (depending on the calculated variance of spots), were considered to contain proteins differentially expressed.

### Protein identification by MALDI-TOF-MS

Spots were excised from 2-DE gels and in-gel triptych digestion was carried out following the procedure described by Shevchenko *et al.*[25]. Matrix Assisted Laser Desorption Ionization - Time of Flight - Mass Spectrometry (MALDI-TOF-MS) analyses were carried out on a Voyager DE PRO mass spectrometer (Applied Biosystems Foster City, CA) operating in positive-ion reflectron mode. Mass spectra were calibrated using as internal standards the monoisotopic peaks of angiotensin (m/z 931.5154) and of adrenocorticotropic hormone (ACTH) fragment 18–39 (m/z 2465.1989) and data were processed using the DataExplorer 5.1 software (Applied Biosystems). Peak lists were obtained by further processing the mass spectra with the Mascot Wizard tool (http://www.matrixscience.com) and manually inspected. Protein identification was achieved by using peak lists for database searches against the NCBInr database using the Mascot software (http://www.matrixscience.com/). Parameters for all searches were as follows: all entries as taxonomic category, trypsin as enzyme, carbamidomethyl as fixed modification for cysteine residues, up to one missing cleavage and up to 50 ppm as mass tolerance. Identified proteins were classified on the basis of their biological functions using the bioinformatic resource KEGG (Kyoto Encyclopedia of Genes and Genomes, http://www.genome.jp/kegg/).

## Results

**Figure 1:**
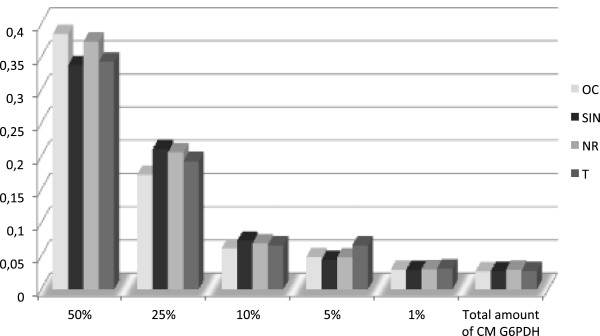
**G6PDH enzymatic activity detected in CM and WCL of the four OC, SIN, NR and T *B. clausi *strains grown aerobically at 37°C in LB medium to stationary growth phase.** CM and bacterial cells were harvested and assayed as described in Materials and Methods. G6PDH activity in CM was compared with that associated with deliberately lysed cell (WCL). The data shown are the means of measurements from three independent experiments. 0.01 units of G6PDH were assayed as a control in each experiment.

**Figure 2:**
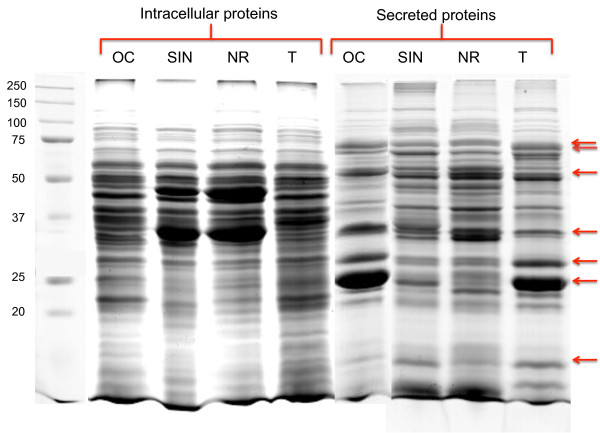
**SDS-PAGE analysis of whole cell proteins (WCL) and CM proteins of the four OC, SIN, NR and T *B. clausii *strains.**  Arrows indicate the CM protein bands which are different from those in WCL. The SDS-PAGE protein profile shown is representative of three independent experiments.

**Figure 3:**
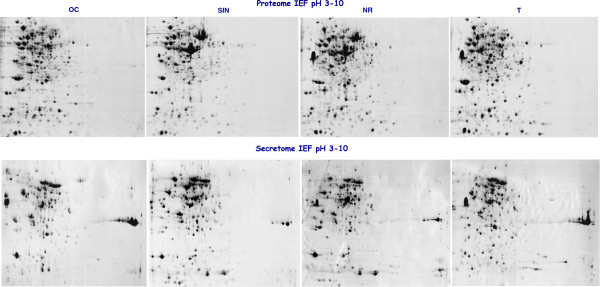
**Comparison of WCL proteins maps and secreted protein maps of the four O/C, SIN, N/R and T *B. clausii *strains at stationary growth phase.**

To rule out the possible presence of intracellular proteins in CM, we measured the amount of the cytosolic marker G6PDH in CM. The total amount of G6PDH enzymatic activity detected in CM was compared with that found to be associated with serial dilutions of WCL samples. As shown in Figure 1, and in Additional file 4: Table S1, the total G6PDH activity detected in CM was less than that found in 1% of the corresponding WCL sample. In addition, an equal amount of CM and WCL proteins of the four strains was analyzed by both SDS-PAGE (Figure 2) and 2-DE (Figure 3), allowing us to directly compare the CM and WCL protein profiles. The electrophoretic patterns thus obtained showed clearly distinct protein profiles of CM and WCL for the four *B. clausii* strains. These results confirmed that the proteins detected in CM did not derive from cell lysis occurring during the cultivation or processing procedures.

### Secretome analysis of the four *B. clausii* strains

Literature data show that the highest levels of protein secretion were observed when bacterial cells were grown in rich medium and during the stationary growth phase [26-29]. In agreement with these findings, our results showed a more abundant and rich repertoire of secreted proteins at stationary growth phase (Additional file 5: Figure S4).

In fact, minor differences in the secretome profiles were observed by comparing the proteomic maps of secreted proteins of bacteria cells at stationary and late exponential growth phase. At early exponential growth phase the total secreted proteins were weakly detectable (Additional file 5: Figure S4). Therefore, comparative proteomic analysis of the overall secretome of the four O/C, SIN, N/R and T *B. clausii* strains, was performed on cells grown at stationary growth phase (24 h).

**Figure 4:**
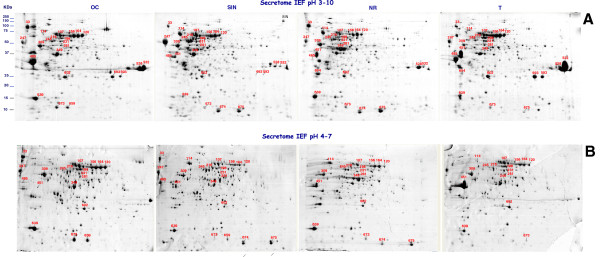
**2-DE analysis of secretomes of the four O/C, SIN, N/R, and T B*. clausii *strains. A**. 2-DE maps of the four *B. clausii*  secretomes from bacterial cells in stationary growth phase separated using pH 3–10 N.L IPG strips. Protein spots showing different intensity in the four strains are indicated by match ID. Proteins contained in these spots were identified by MALDI-TOF-MS and database searches (Table 1). **B**. 2-DE maps of the four *B. clausii*  secretomes from bacterial cells in stationary growth phase in the 4–7 pI range. Protein spots showing different intensity in the four strains, are indicates by numbers referred to Figure 4A.

The four strains secreted a large array of proteins, distributed over a wide range of pI values and molecular masses. In Figure 4, representative gels of secretome of the four strains, obtained in the pI range 3–10 and 4–7 are shown. The overall position of protein spots was similar in the gels of the four strains.

The number of spots observed in the gels was similar in the four classes without significant differences (431 ± 38 in OC, 453 ± 26 in SIN, 410 ± 30 in NR and 455 ± 42 in T). Also the percentage of matches between gels from the same class (intra-class) and from the four different classes (inter-classes) was similar (around 60%). Some spots were not considered for analysis because they were present only in a subset of gels and exhibited variability. Only protein spots systematically present in at least two of tree gels of each class, were considered in the analysis

Among this group of spots, only those showing a different mean intensity in the gels from the four strains with a p < 0.05, applying the restrictive two tailored Student’s *t*-test, were considered to contain proteins differentially expressed. Using this constrained statistical analysis, unexpected pronounced variations in mean intensity of 26 spots, marked in Figure 4, were revealed.

Proteins contained in these spots were identified applying the Peptide Mass Fingerprinting (PMF) strategy and results are summarized in Table 1.

**Table 1:** **Secreted proteins differentially expressed by the four probiotic strains of *B. clausii*, as identified by PMF strategy**

**Functional classification**	**Match ID**^**a**^	**Gene**^**b**^	**Protein name**	**Accession Number (NCBInr)**	**Kegg Entry**^**c **^**(organism Bcl)**	**M.W.**^**d **^**(kDa)**	**pI**^**e**^	**Mascot Score**	**Number of matched peptides**	**Sequence Coverage(%)**	**Localization**	**References**
**Carbohydrate metabolism**	309	*eno*	Phosphopyruvate hydratase (Enolase)	gi|56964781	ABC3017	46226	4.66	294	19	58	Cytoplasm. Secreted. Cell surface without export signal	Antelmann et al. *Genome Res.* 2001, 11:1484
	382	*adhA*	Alcohol dehydrogenase	gi|56961828	ABC0046	37979	5.26	230	15	69	Cytoplasm.	
	107	*lpd*	Dihydrolipoamide dehydrogenase	gi|56964182	ABC2452	49579	5.25	261	21	47	Membrane. Extracellular Membrane bound	Engels et al. BBA. 1997, 1340:33
	207	*lpd*	Dihydrolipoamide dehydrogenase	gi|56964182	ABC2452	49579	5.19	207	18	67	Membrane. Extracellular Membrane bound	Engels et al. BBA. 1997, 1340:33
			Succinate-semialdehyde dehydrogenase	gi|56962111	ABC0331	51161	5.23	109	15	44		
	216	*acsA*	Acetyl-CoA synthetase	gi|56964524	ABC2760	64388	5.24	293	26	50		
	439	*mdh*	Malate dehydrogenase	gi|56964478	ABC2713	33576	5.16	119	10	62	Cytoplasm Secreted without export signal	Antelmann et al *Genome Res.* 2001, 11:1484
			Sugar Phosphate isomerase/epimerase	gi|56962569	ABC0795	36078	5.18	227	15	83		
	231	*pgi*	Glucose-6-phosphate isomerase	gi|56964670	ABC2906	50356	5.25	378	28	87		
**Peptides/Nickel transport system**	33		Oligopeptide ABC transporter substrate-binding protein	gi|56964179	ABC2414	65042	4.07	271	24)	55	Secreted with export signal	Antelmann et al. *Genome Res.* 2001, 11:1484
			Oligopeptide ABC transporter substrate-binding protein	gi|56964854	ABC3090	68534	4.09	257	25	50	Secreted with export signal	Antelmann et al. *Genome Res.* 2001, 11:1484
**Environmental information Processing Membrane transport**	247	*mntA*	Mn2+/Zn2+ ABC transporter substrate-binding protein	gi|56965719	ABC3961	34305	3.78	65	4	27	Secreted with export signal	Antelmann et al. *Genome Res.* 2001, 11:1484
**Proteases/Peptidases**	532	*aprE*	Chain A, Alkaline M-Protease Form I Crystal Structure	gi|56966974	ABC0761	26707	9.30	110	8	40	Secreted without export signal	Antelmann et al. *Genome Res.* 2001, 11:1484
	593	*aprE*	Chain A, Alkaline M-Protease Form I Crystal Structure	gi|56966974	ABC0761	26707	9.30	147	8	40	Secreted. without export signal	Antelmann et al. *Genome Res.* 2001, 11:1484
	693	*aprE*	Chain A, Alkaline M-Protease Form I Crystal Structure	gi|56966974	ABC0761	26707	9.30	97	5/6	27	Secreted without export signal	Antelmann et al. *Genome Res.* 2001, 11:1484
	245	*ampS*	Aminopeptidase	gi|56963895	ABC2130	45195	5.06	197	18	46	Secreted with export signal	Wei Wang et. al. Proteome Science. 2006, 4:19
**Antioxidant defence**	120	*cat*	Catalase	gi|56963055	ABC1283	55939	5.52	360	31	82	Secreted without export signal	Antelmann et al. *Genome Res.* 2001, 11:1484
	156	*cat*	Catalase	gi|56963055	ABC1283	55939	5.52	375	28	67	Secreted without export signal	Antelmann et al *Genome Res.* 2001, 11:1484
	164	*cat*	Catalase	gi|56963055	ABC1283	55939	5.52	295	20	56	Secreted without export signal	Antelmann et al. *Genome Res.* 2001, 11:1484
	694		2-cys Peroxiredoxin	gi|56964193	ABC2428	20402	4.56	143	9		Secreted	Min-Ho Cho et al. Molecular Biochemical Parasitology, 2005, 143:80
	602	*sodA*	Manganese Superoxide dismutase	gi|56963480	ABC3961	22330	5.41	132	7	59	Secreted without signal peptides	Antelmann et al. *Genome Res.* 2001, 11:1484
**Energy metabolism**	485		Carbonic anhydrase	gi|56964946	ABC3182	29268	4.37	98	6	22	Secreted	Oviya M et al. Prep Biochem Biotechnol. 2012 42:462
**RNA synthesis**	281		Transcriptional regulator	gi|56962515	ABC0741	47467	5.35	341	26	77	Secreted with signal peptides	H Tjalsma et al. Microbiology and Molecular Biology reviews, 2004, 68:207
**Structural protein**	451	*hag*	Flagellin protein	gi|56965461	ABC3699	32042	4.52	121	11	34	Cytoplasm. Secreted without export signal	Antelmann et al. *Genome Res.* 2001, 11:1484
**Cell-Wall metabolism**	528	*cwlC*	Acetylmuramoyl-L-Alanine amidase	gi|56962701	ABC0927	27617	8.89	165	12	54	Cell surface	Nouwens et al. *Microbiology 2003, 149: 1311*
**Environmental Information Processing**	528	*resD*	Two-component response regulator	gi|56963600	ABC1835	27512	5.17	95	9	40		
**Molecular Chaperones**	114	*groEL*	molecular chaperone GroEL	gi|56962656	ABC0882	57240	4.76	33		66	Secreted	Beck et al. FEMS Microbiol Lett 2009, 297:6166
	639		Hypothetical protein ABC0920	gi/56962694		14960	4.40	109	6	73	Secreted	Antelmann et al. *Genome Res.* 2001 11: 1484
	673		Hypothetical protein ABC2092	gi|56963857	ABC2092	13501	5.41	130	10	84	Secreted	Antelmann et al. *Genome Res.* 2001 11: 1484
	659		Hypothetical protein ABC2108	gi|56963873	ABC2108	13099	5.55	120	8	59	Secreted	Antelmann et al. *Genome Res.* 2001 11: 1484

*a.* ID Spot numbers refer to Figure 4.

b. Gene.

c. Kegg Entry. Organism: *B. clausii* KSM-K16 (Bcl).

*d.* Theoretical molecular weight (kDa).

*e.* Theoretical isoelectric point.

The secreted proteins differentially expressed by the four stains, could be classified on the basis of their biological function among several categories.

(i.) Eight enzymes related to carbohydrate metabolism: dihydrolipoamide dehydrogenase (Lpd), spot 107, expressed at higher level in strains SIN, and T; succinate semialdeyde dehydrogenase (Ssdh), spot 207 which comigrated with dihydrolipoamide dehydrogenase; acetyl CoA synthetase (AcsA), spots 216 overexpressed in SIN strain; glucose-6-phosphate isomerase (Pgi), spot 231, present in higher amount in SIN strain; enolase (Eno), spot 309, underexpressed in T strain; alcohol dehydrogenase (AdhA), spot 382, overexpressed in SIN and NR strains and malate dehydrogenase (Mdh) and sugar phosphate epimerase, spot 439, which co-migrated.

(ii.) Three proteins, substrate-binding component of various transport systems: two oligopeptide ABC transporters (ABC2414 and ABC3090) which co-migrated spots 33, expressed at higher level in strains OC and NR; Mn^2+^/Zn^2+^ ABC transporter, spot 247, overexpressed in SIN strain.

(iii.) Two proteases: chain A, alkaline protease (AprE), spots 532, 593 and 693, secreted at more abundant level in strains OC and T and aminopeptidase (AmpS), spot 245, overexpressed in SIN strain.

(iv.) Four proteins involved in detoxification: carbonic anhydrase, spot 485, was detected at significantly abundant level in strains OC and T; 2-Cys peroxiredoxin, spot 694, detected in strain NR*;* manganese superoxide dismutase (SodA), spot 602, expressed at higher level in strain OC and at very low level in strain T and catalase (Cat), spots 120, 156 and 154, which migrated as pearl chains, probably due to post translational modifications, expressed at higher level in OC and NR strains.

(v.) The molecular chaperone GroEL, spot 114, overexpressed in strains SIN and NR.

(vi.) A structural flagella-related protein flagellin (Hag), spot 451, significantly more abundant in strains NR and T.

(vii.) One transcriptional regulator, spot 281, overexpressed in SIN strain.

(viii.) A two-component response regulator, spot 674, overexpressed in SIN and NR strains.

(ix.) Three proteins of unknown function: hypothetical protein ABC 0920, spot 639; hypothetical protein ABC2092 spot 659; hypothetical protein ABC2108, spot 673.

**Figure 5:**
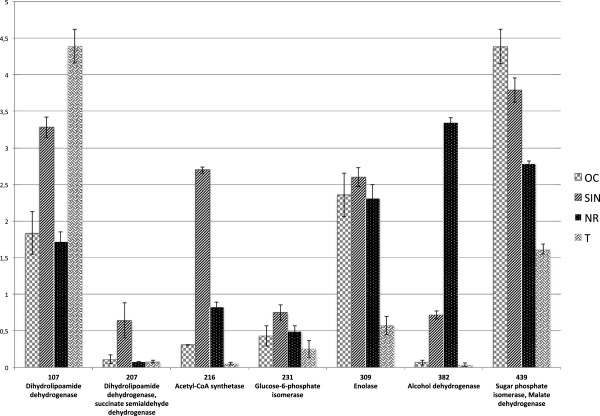
**Semiquantitative expression profile, in the four strains, of identified proteins belonging to carbohydrate metabolism.** Each spot is indicated with a match ID. The error bars (SEM) are indicated.

**Figure 6:**
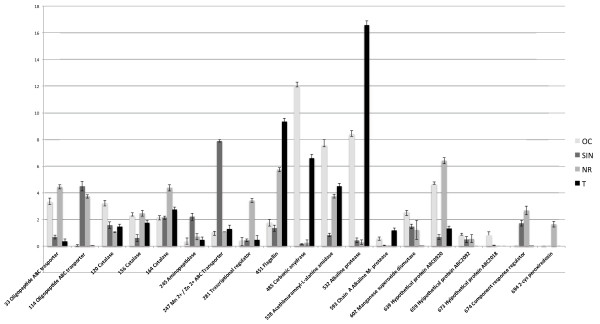
**Expression profile in the four strains of other identified proteins.** Each spot is indicated with a match ID. The error bars (SEM) are indicated.

These results are shown in Figures 5, 6 and summarized in Table 1 and Additional file 6: Table S2.

Most of the identified extracellular proteins migrated on the 2D gels at a position that was in good agreement with their calculated molecular weight and isoelectric point. Spots ID 593 and 693, containing chain A alkaline protease, did not run at the estimated position. Most likely, this was due to proteolytic processing upon export from the cytoplasm. The two major degradation products of alkaline protease were both detected in the two OC and T strains, which secreted high level of proteases (Figure 4).

All the identified proteins were from *B. clausii* KSM-K16 thus confirming the identification accuracy and the similarity among the *B. clausii* KSM-K16 and the four strains analyzed in this work.

## Discussion

The four OC, SIN, NR and T, *B clausii* strains, are used in a pharmaceutical preparation as probiotics for oral bacteriotherapy and bacterioprophylaxis of gastrointestinal disorders sometimes combined with the antibiotic therapy.

In the present study, for the first time, proteomics was applied to the secretome analysis of the four isogenic strains. Each strain secreted a large repertoire of proteins into the extracellular environment that comes into direct contact with host compartments and can mediate host-bacteria interactions and probiotic effects.

Although the genomes of the four *B clausii* strains are very similar, as their life cycles, biology, and protein expression profiles [4], the comparative proteomic analysis revealed, for the first time, unexpected variations in the expression level of some secreted proteins between the four strains at stationary growth phases and shed new light on the specific secretome features of each strain. The reported results highlighted phenotypic differences between these strains, and suggested that regulatory circuits were differently active in determining secretion levels of some proteins.

In particular, OC strain expressed at higher level enolase, carbonic anhydrase, acetylmuramoyl-L-alanine amidase and alkaline protease; SIN strain overexpressed enolase, GroEL and two oligopeptide ABC transporters; NR strain overexpressed enolase, GroEL, catalase, flagellin protein, two oligopeptide ABC transporters and a transcriptional regulator factor; T strain expressed the flagellin protein and alkaline protease at very high level and overexpressed acethilmuramoyl-L- amidase (Figure 5, Figure 6 and Additional file 6: Table S2).

Many of the proteins identified in the present study were already reported to be involved in the molecular mechanisms of probiotic actions, in particular in the adaptation and colonization processes of the human gastrointestinal tract (GIT) as well as in immunomodulatory mechanisms.

The presence of cytoplasmic proteins as secretome components of the four *B. clausii* strains is not surprising. A growing list of prokaryotic and eukaryotic proteins shows, in fact, intracellular and extracellular dual localization [30-32]. These cytoplasmic proteins are displayed on the cell surface of a wide spectrum of Gram-positive and Gram-negative bacteria [32,33], where they acquire a secondary ‘moonlighting’ function [34] important in bacteria-host interactions.

Some of these proteins have been shown to have human plasminogen/fibronectin/mucus binding activity and thus may be involved in adhesion and colonization of GIT. Our proteomic analysis identified two potential plasminogen-binding proteins: enolase, underexpressed in T strain and flagellin overexpressed in NR and T strains.

The surface-associated α-enolase of several, mainly Gram-positive pathogenic bacteria, was identified as a major protein component with plasminogen binding capacity, thus enhancing the proteolytic plasmin activity important in the pathogenesis process [33]. The mechanisms used by our strains to attach to intestinal mucosa might mimic those of pathogens, therefore mediating the exclusion of enteropathogenic bacteria by competing for binding sites [35].

Strains SIN and NR secreted very high level of the molecular chaperone GroEL which has been reported to act as a moonlighting protein, being part of the extracellular proteome of several probiotic species [36]. GroEL was also shown to have a key role in the adhesion mechanism of probiotics, being able to bind to mucins and human intestinal epithelial cells [37,38]. Moreover, this protein is involved in immunomodulation, stimulates interleukin-8 secretion in macrophages and HT29 cells in CD14-dependent pro-inflammatory response and mediates the aggregation of the gastric pathogen *H. pylori*[39].

Flagellin protein has been recently recognized to be a potent activator of intestinal epithelial pro-inflammatory gene expression [34,40]. It has been suggested that the stimulation induced by flagellin secreted by probiotics would not reach the threshold level necessary for the induction of a pro-inflammatory response, producing instead an activation of the immune system through the production of IL-8 and “human βdefensin” (2 hBD-2) [41] which counteract bacterial adherence and invasion [42].

Transporter substrate-binding proteins and proteins involved in cell wall metabolism, also identified in the present study, have been found to have potential probiotic activities [43]. ABC transporters represent a major class of secreted proteins in Gram-positive bacteria [38], having a key role in nutrient intake and chemotaxis and in antibiotic and antifungal resistance with considerable medical relevance [44]. We have identified two ABC transporter substrate-binding proteins expressed at higher level in the secretome of strains NR and OC (Figure 4, Figure 6).

The cell wall-metabolizing protein acetylmuramoyl-L-alanine amidase, which catalyzes turnover and degradation of peptidoglycan [45], was found to be overexpressed in OC strain.

All the four *B. clausii* strains secreted at different level catalase, manganese superoxide dismutase and 2-cys peroxiredoxin, enzymes of the redox system (Figure 4, Figure 6). Recently it has been demonstrated that genetically engineered lactic acid bacteria producing antioxidant enzymes, could be used to prevent or decrease the severity of certain intestinal pathologies caused by reactive oxygen species; catalase and superoxide dismutase were also evaluated from an immune stimulating (cytokine producing) point of view [46]. Recently, peroxiredoxins received considerable attention as a new and expanding family of thiol-specific antioxidant proteins through their peroxidase activity [47]. As the anti-oxidant potential of probiotics is currently the focus of attention, the four strains that differentially expressed these enzymes, could be explored as prospective antioxidants to manage oxidative stress.

The four strains expressed differentially a cytoplasm membrane associated protein dihydrolipoamide dehydrogenase (Figure 4, Figure 6). It has been suggested that this enzyme might have a fundamental role in membrane processes such as transport of solutes into and out of the cell [48].

Strain NR secreted higher amount of aminopeptidase. This protein participates in a wide range of biological processes, from protein maturation or degradation to cell cycle control. The higher secretion of aminopeptidase probably indicates a higher rate in recycling small peptides that become available through the degradation of proteins/peptides in the culture medium, and serve as amino acid sources to sustain cell growth under nutrient limitation. They can, also, play signaling roles in the initiation of different cellular processes such as competence development and sporulation [20].

Our comparative proteomic analysis identified proteins differentially expressed in the secretome of the four *B. clausii* indicating that each strain is characterized by a specific secretion pattern, despite their genotypic similarity, which can contribute to specific interaction with host cells. It is conceivable that the clinical effects of oral administration of this preparation are contributed by the sum of the specific probiotic properties associated with each strain. The proteins identified in the present study open the way to further studies aimed at investigating their claimed probiotic function.

## Conclusions

In this study comparative proteomics was used to analyze the secretome of four isogenic *B. clausii* probiotic strains. Results provided an overview of secretome patterns of the four strains and showed clear different features of protein secretion, indicating strain specific profile of secretomes. Differential expression of proteins with claimed probiotic activity could reflect specific ability of each strain to act as probiotics.

Secretome analyses could then represent a powerful tool to identify specific proteins, which may serve as bacterial biomarkers for the selection of strains with the best probiotic potential.

## Abbreviations

IPG: Immobilized pH gradient; ID: Identification number; LB: Luria Bertani; CHAPS: [(3-cholamidopropyl) dimethylammonio]-1-propanesulfonate; DTT 1: 4-dithio-DLthreitol; PMSF: Phenylmethylsulfoniyl fluoride; SDS: Sodium dodecyl sulphate; CM: Culture medium; G6PDH: Glucose 6-phosphate dehydrogenase.

## Competing interests

The authors declare that they have no competing interests.

## Authors’ contributions

RL conceived of the study and its experimental design, carried out the experimental proteomic study and wrote the manuscript. RAS and MFM performed MS experiments and bioinformatics processing of MS data. AA cultured the B. clausii strains. AMS and AG participated in the coordination and helped to manuscript preparation. SP participated in the experiment design and revised the manuscript critically for important intellectual content. All authors read and approved the final manuscript.

## Supplementary Material

Additional file 1: Figure S1 Growth curve of OC, SIN, NR and T *B. clausii*  strains. Cell growth was quantified in LB medium by measuring the optical density at 595 nm. Click here for file

Additional file 2: Figure S2 DE. Reproducible gel images of proteins secreted by the four strains. Equivalent amounts (250 μg) of secreted proteins, from cells in the stationary growth phase were separated, using linear pH ranges 3–10 IPG strips. SDS-PAGE was performed with 12.5% acrylamide. Gels were stained with Colloidal Coomassie brilliant blue G-250. Click here for file

Additional file 3: Figure S3 2-DE maps of the LB medium without cell inoculation, incubated to 37°C for 24 h. Equal amount of TCA-precipitated volume of CM with and without bacteria cells, at stationary growth phase, was subjected to 2-DE analysis, using linear pH ranges 3–10 IPG strips. SDS-PAGE was performed with 12.5% acrylamide. Gels were stained with Colloidal Coomassie brilliant blue G-250. Click here for file

Additional file 4: Table S1 G6PDH enzymatic activity of CM and WCL of the four OC, SIN, NR and T *B. clausii* strains. The four strains were grown aerobically at 37°C in LB medium to stationary growth phase. Cells and CM were harvested and assayed as described in Materials and Methods. G6PDH activity was assayed by following the reduction of NADP at 340 nm. The reaction was started by addition of either WCL or cell-free CM. The final concentrations in the reaction mixture were Glu- 6P, 1.0 mM; NADP, 0.4 mM; MgCI_2_, 6.9 mM; and Tris-HC1, pH 7.8, 50 mM. Values reported are the mean of three determinations. 0.01 units of G6PDH were assayed as a control in each experiment. *One activity unit is defined as the amount of enzyme that catalyzes the transformation of 1 mmol of substrate per min under the conditions of the assay. Click here for file

Additional file 5: Figure S4 2-DE analysis of secretomes of cell in **a**) early exponential growth phase, **b**) late exponential growth phase, **c**) stationary growth phase. Equivalent amounts (250 μg) of secreted proteins, were separated, using NL pH ranges 3–10 IPG strips (GE Healthcare). SDS-PAGE was performed with 12.5% acrylamide. Gels were stained with Colloidal Coomassie brilliant blue G-250. Click here for file

Additional file 6: Table S2 Image analysis of 2-DE maps of secretome of the four *B. clausii* strains: Spots showing different mean intensity in the four maps are listed , together with the corresponding identified proteins. Reported values are the mean + SD of spot volumes (% vol) observed in the OC, SIN, NR, and T, proteomic electropherograms examined for each of three independent cultivation taken at the stationary (24 h) growth phase. Proteins are listed according to their respective match spot identification numbers (ID). The match ID is referred to those reported in Figure 4. Click here for file
