# Supplementary figures and images for: Comparative secretome analysis of four isogenic Bacillus clausii probiotic strains
Source: Proteome Sci. 2013 Jul 1;11:28. doi: 10.1186/1477-5956-11-28 (PMC3716886; doi:10.1186/1477-5956-11-28)

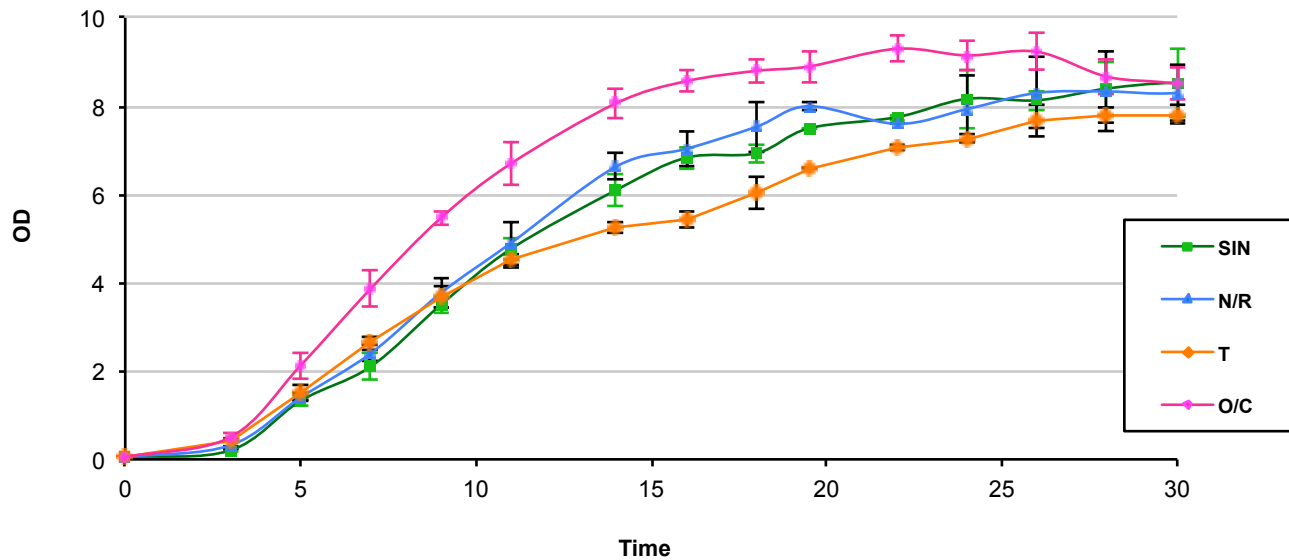

Supplement: Additional file 1: Figure S1 — Growth curve of OC, SIN, NR and T B. clausii strains. Cell growth was quantified in LB medium by measuring the optical density at 595 nm. [file 1477-5956-11-28-S1.pdf]

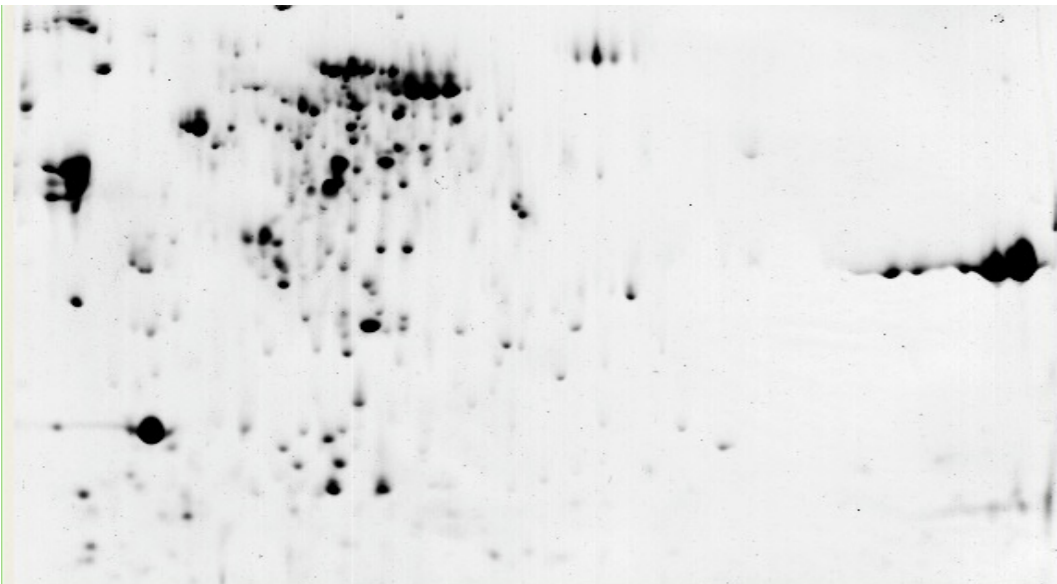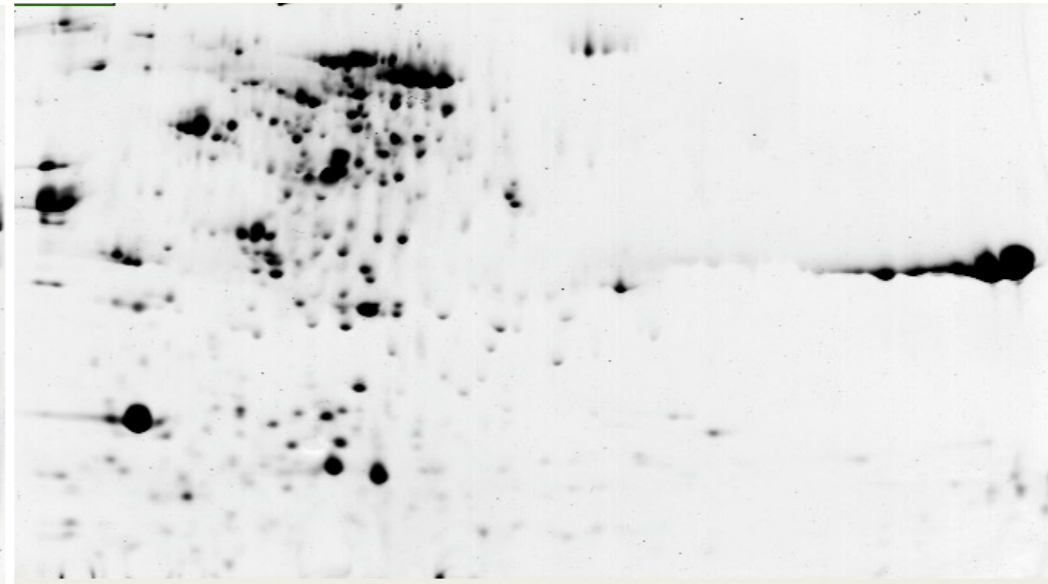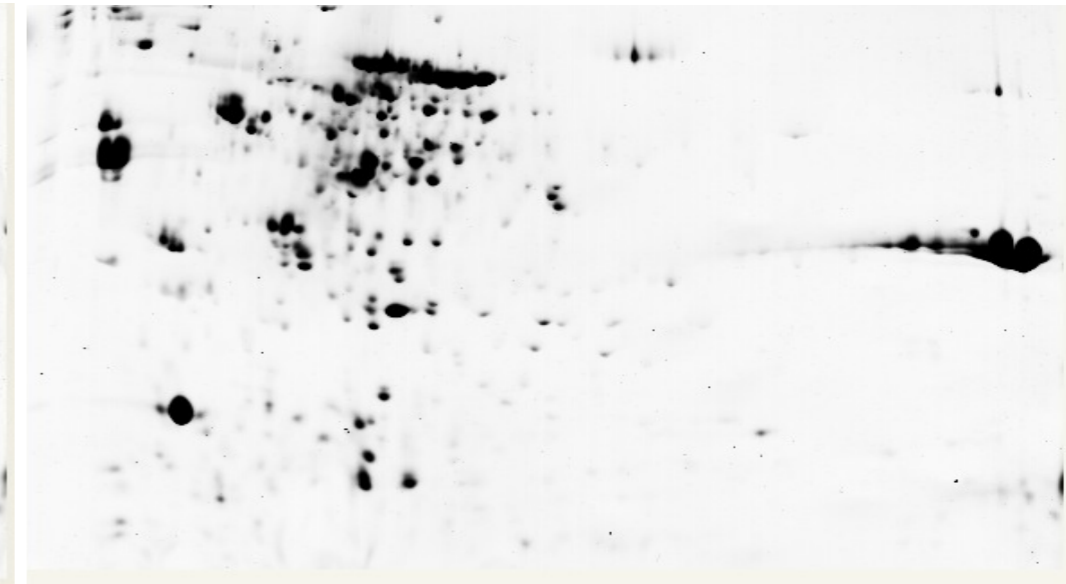

O/C

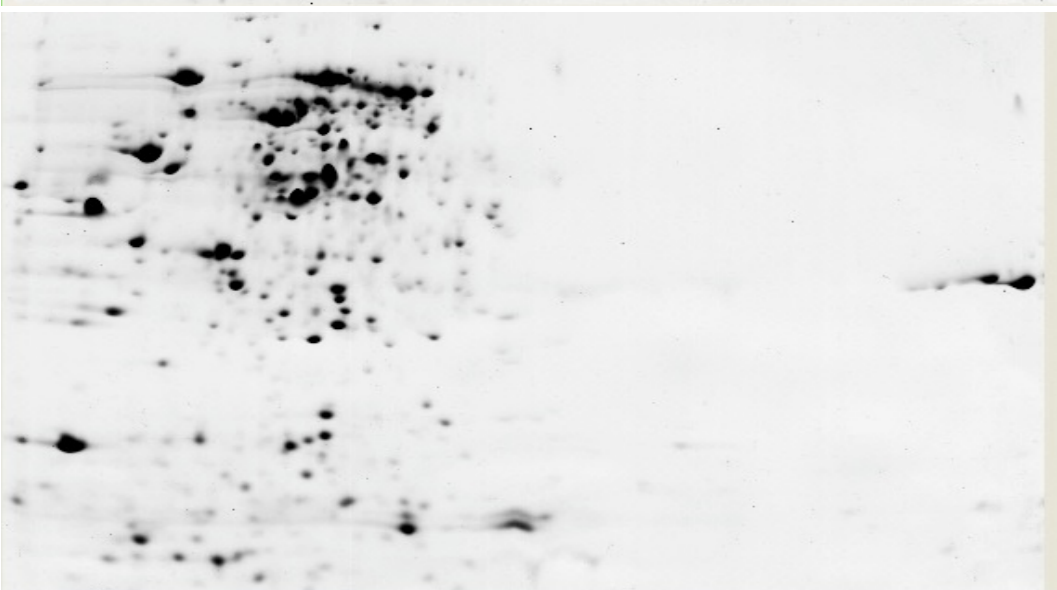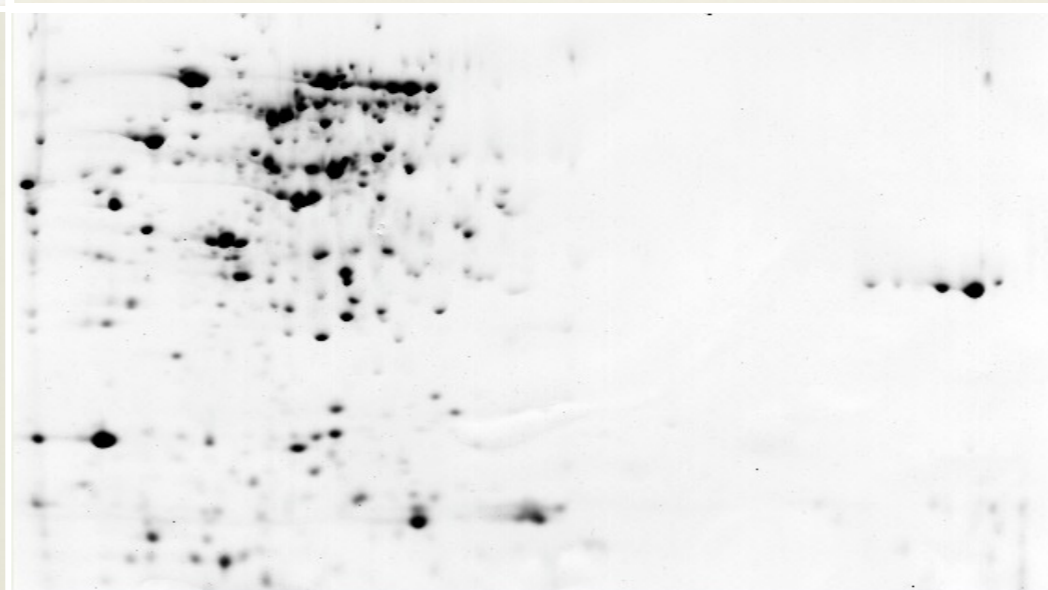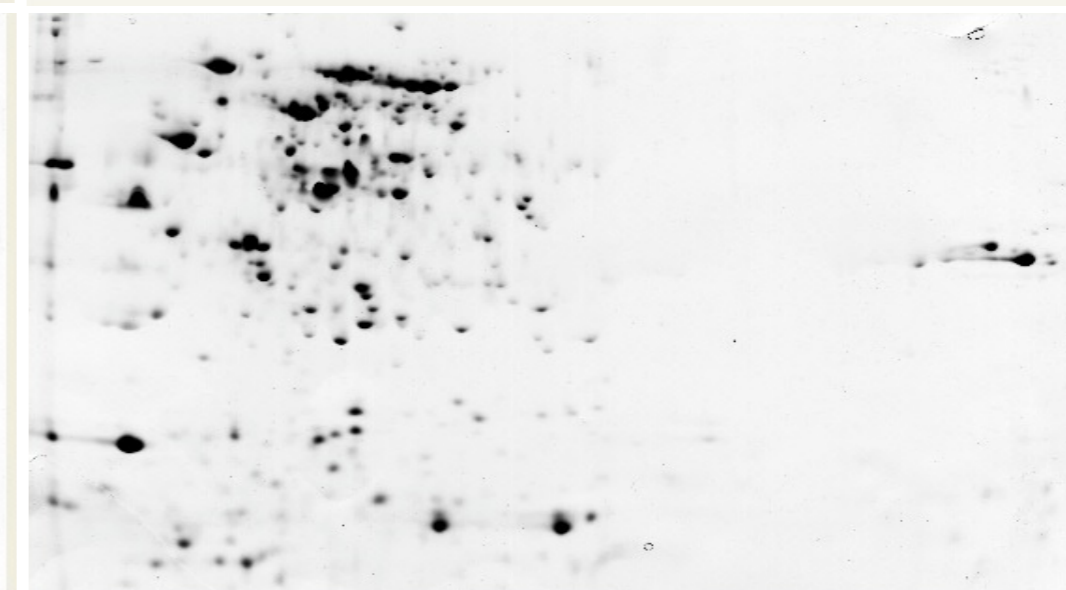

SIN

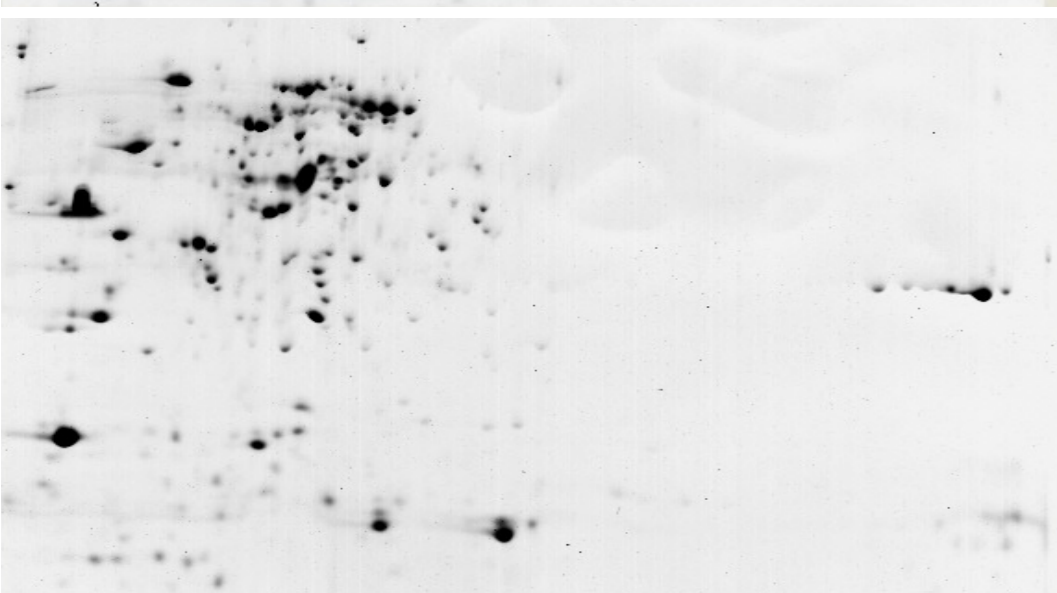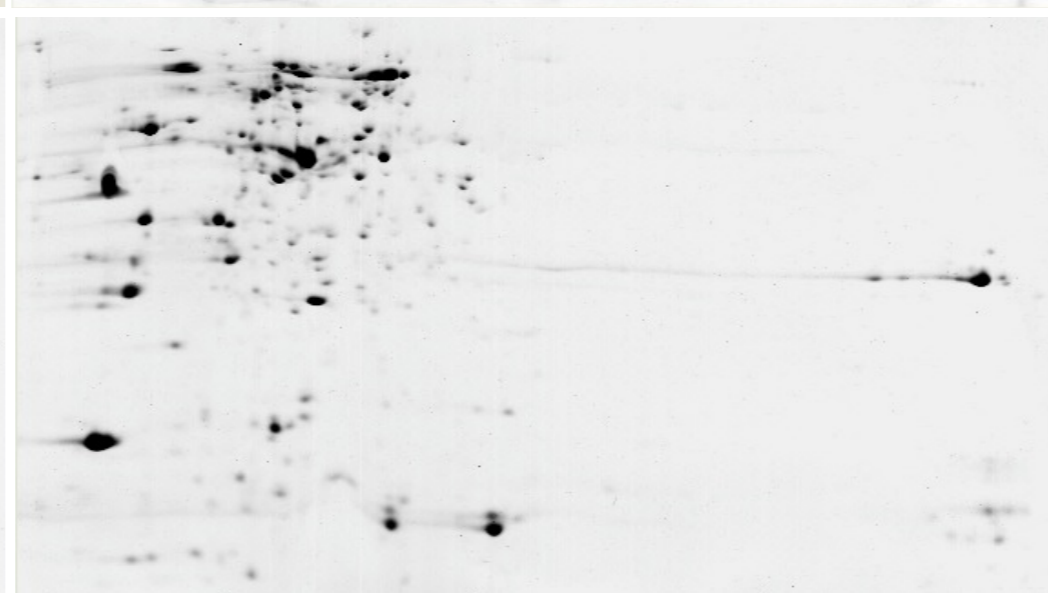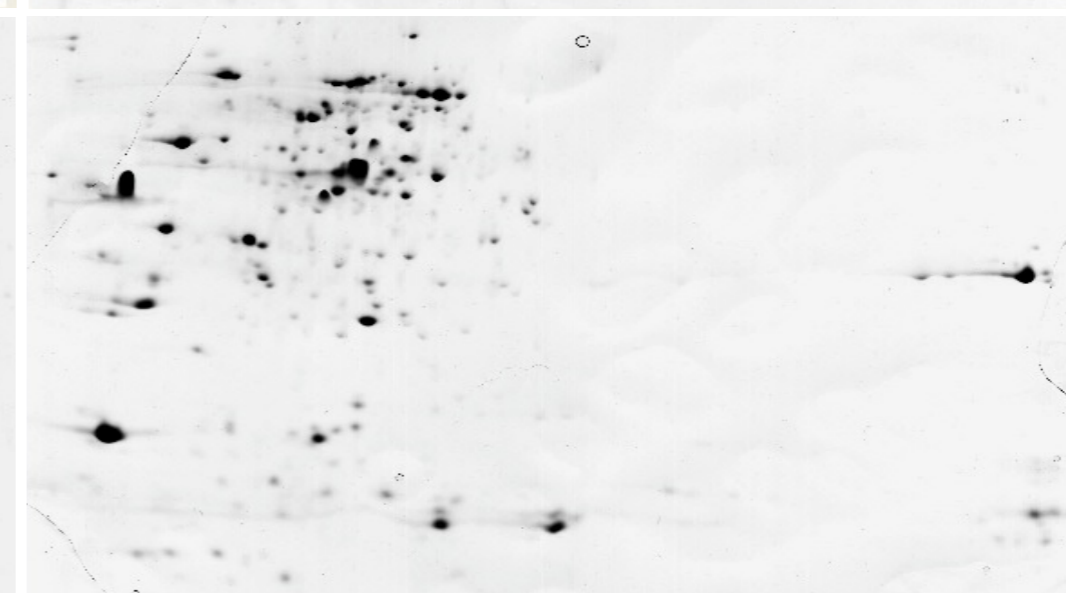

N/R

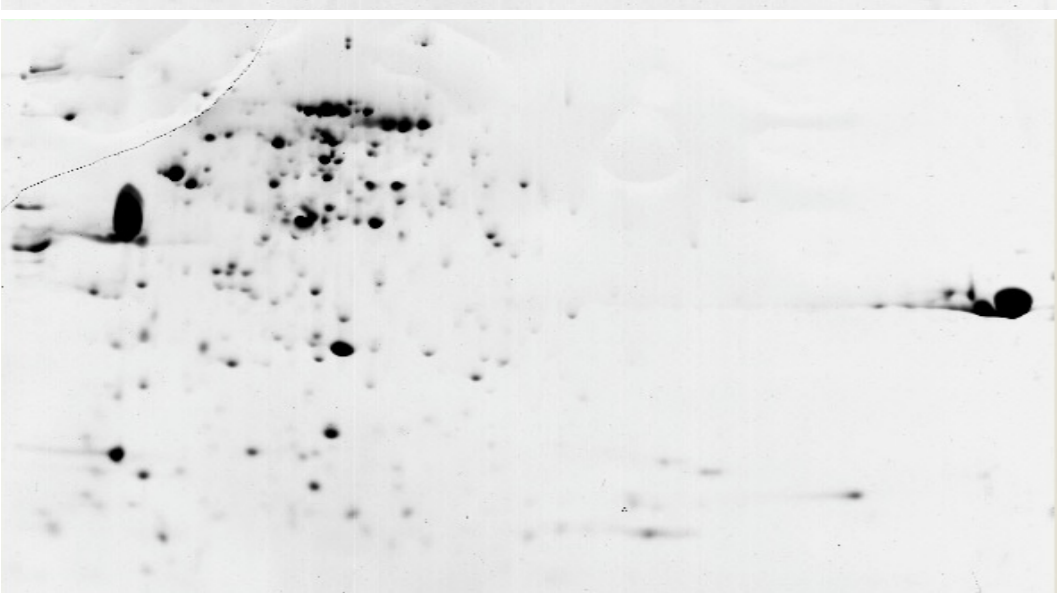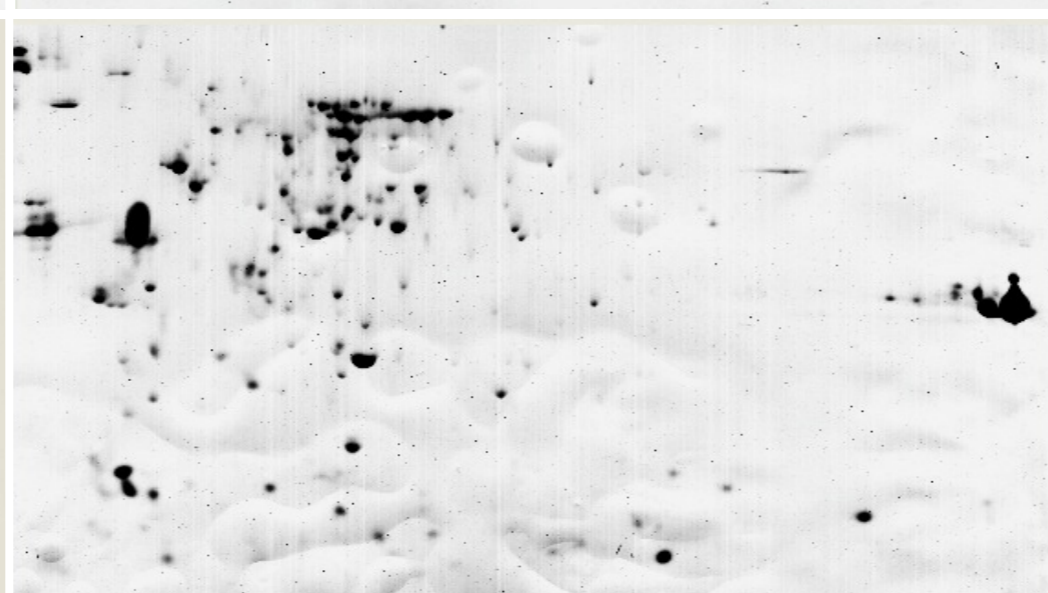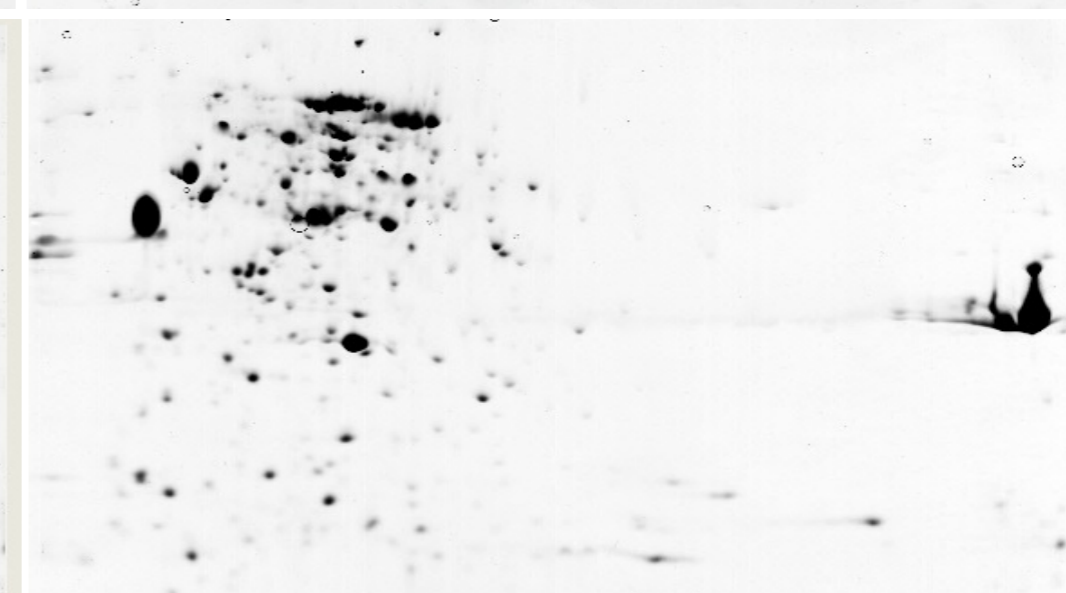

T

Supplement: Additional file 2: Figure S2 — DE. Reproducible gel images of proteins secreted by the four strains. Equivalent amounts (250 μg) of secreted proteins, from cells in the stationary growth phase were separated, using linear pH ranges 3–10 IPG strips. SDS-PAGE was performed with 12.5% acrylamide. Gels were stained with Colloidal Coomassie brilliant blue G-250. [file 1477-5956-11-28-S2.pdf]

KDa

250 —  
150 —  
100 —  
75 —  
50 —  
37 —  
25 —  
20 —  
15 —  
10 —

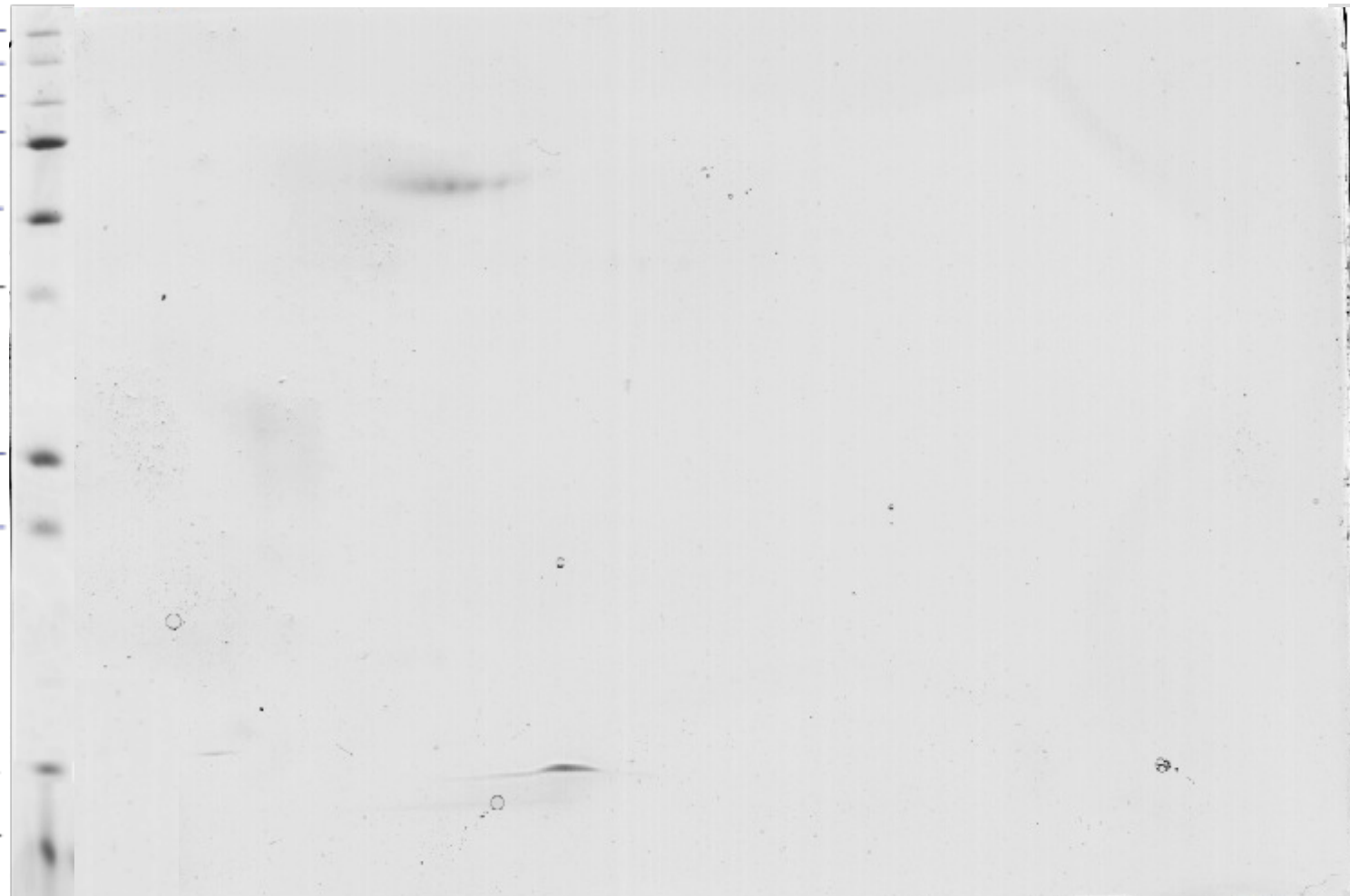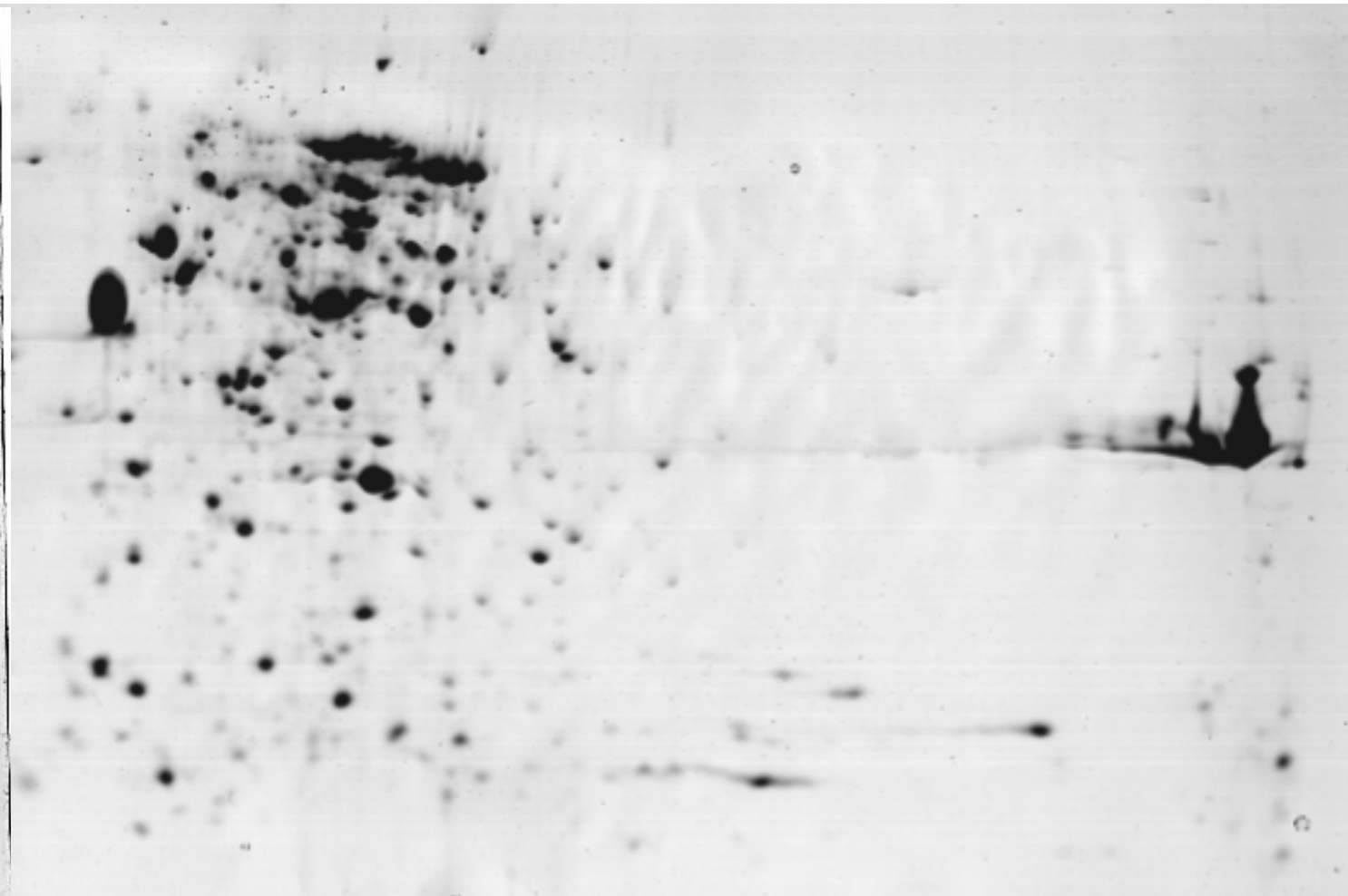

Supplement: Additional file 3: Figure S3 — 2-DE maps of the LB medium without cell inoculation, incubated to 37°C for 24 h. Equal amount of TCA-precipitated volume of CM with and without bacteria cells, at stationary growth phase, was subjected to 2-DE analysis, using linear pH ranges 3–10 IPG strips. SDS-PAGE was performed with 12.5% acrylamide. Gels were stained with Colloidal Coomassie brilliant blue G-250. [file 1477-5956-11-28-S3.pdf]
