# Supplementary material for: Comparative secretome analysis of four isogenic Bacillus clausii probiotic strains
Source: Proteome Sci. 2013 Jul 1;11:28. doi: 10.1186/1477-5956-11-28 (PMC3716886; doi:10.1186/1477-5956-11-28)
Supplement: Additional file 4: Table S1 — G6PDH enzymatic activity of CM and WCL of the four OC, SIN, NR and T B. clausii strains. The four strains were grown aerobically at 37°C in LB medium to stationary growth phase. Cells and CM were harvested and assayed as described in Materials and Methods. G6PDH activity was assayed by following the reduction of NADP at 340 nm. The reaction was started by addition of either WCL or cell-free CM. The final concentrations in the reaction mixture were Glu- 6P, 1.0 mM; NADP, 0.4 mM; MgCI2, 6.9 mM; and Tris-HC1, pH 7.8, 50 mM. Values reported are the mean of three determinations. 0.01 units of G6PDH were assayed as a control in each experiment. *One activity unit is defined as the amount of enzyme that catalyzes the transformation of 1 mmol of substrate per min under the conditions of the assay. [file 1477-5956-11-28-S4.docx]

**Tab. S. I**

|  | **G6PDH Enzymatic activity** | | | |
| --- | --- | --- | --- | --- |
|  | OC | SIN | NR | T |
| **Total amaunt of CM G6PDH** | 0,027± 0,005 | 0,028 ± 0,0055 | 0,029 ± 0,0024 | 0,027 ± 0,003 |
| **50% Lysed bacteria cells** | 0,385 ± 0.062 | 0,338 ± 0,0056 | 0,373 ± 0,050 | 0,343 ± 0,06 |
| **25% Lysed bacteria cells** | 0,173 ± 0,018 | 0,212 ± 0,019 | 0,206 ± 0.03 | 0.193 ± 0,0015 |
| **10% Lysed bacteria cells** | 0.062 ± 0.0081 | 0,073 ± 0,0031 | 0,0696 ± 0,007 | 0,066 ± 0,006 |
| **5% Lysed bacteria cells** | 0,049 ± 0,0059 | 0,045 ± 0,0032 | 0,048 6± 0,0065 | 0,056 ± 0,024 |
| **1% Lysed bacteria cells** | 0,03 ± 0,0098 | 0,029 ± 0,0055 | 0,031 ± 0,0032 | 0,031 ± 0,0035 |
| G6PDH | 0,040 ± 0,0015 | 0,041 ± 0,0025 | 0,039 ± 0,0007 | 0,035 ± 0,0026 |

**Tab. S. 1.**  **G6PDH enzymatic activity of CM and WCL of the four OC, SIN, NR and T** *B. clausii* **strains.**

The four strains were grown aerobically at 37°C in LB medium to stationary growth phase. Cells and CM were harvested and assayed as described in Materials and Methods.

G6PDH activity was assayed by following the reduction of NADP at 340 nm*.* The reaction was started by addition of either WCL or cell-free CM. The final concentrations in the reaction mixture were Glu- 6P, 1.0 mM; NADP, 0.4 mM; MgCI_2_, 6.9 mM; and Tris-HC1, pH 7.8, 50 mM. Values reported are the mean of three determinations.

0.01 units of G6PDH were assayed as a control in each experiment.

*One activity unit is defined as the amount of enzyme that catalyzes the transformation of 1 mmol of substrate per min under the conditions of the assay.
