# Supplementary material for: Comparative secretome analysis of four isogenic Bacillus clausii probiotic strains
Source: Proteome Sci. 2013 Jul 1;11:28. doi: 10.1186/1477-5956-11-28 (PMC3716886; doi:10.1186/1477-5956-11-28)
Supplement: Additional file 5: Figure S4 — 2-DE analysis of secretomes of cell in a) early exponential growth phase, b) late exponential growth phase, c) stationary growth phase. Equivalent amounts (250 μg) of secreted proteins, were separated, using NL pH ranges 3–10 IPG strips (GE Healthcare). SDS-PAGE was performed with 12.5% acrylamide. Gels were stained with Colloidal Coomassie brilliant blue G-250. [file 1477-5956-11-28-S5.pdf]

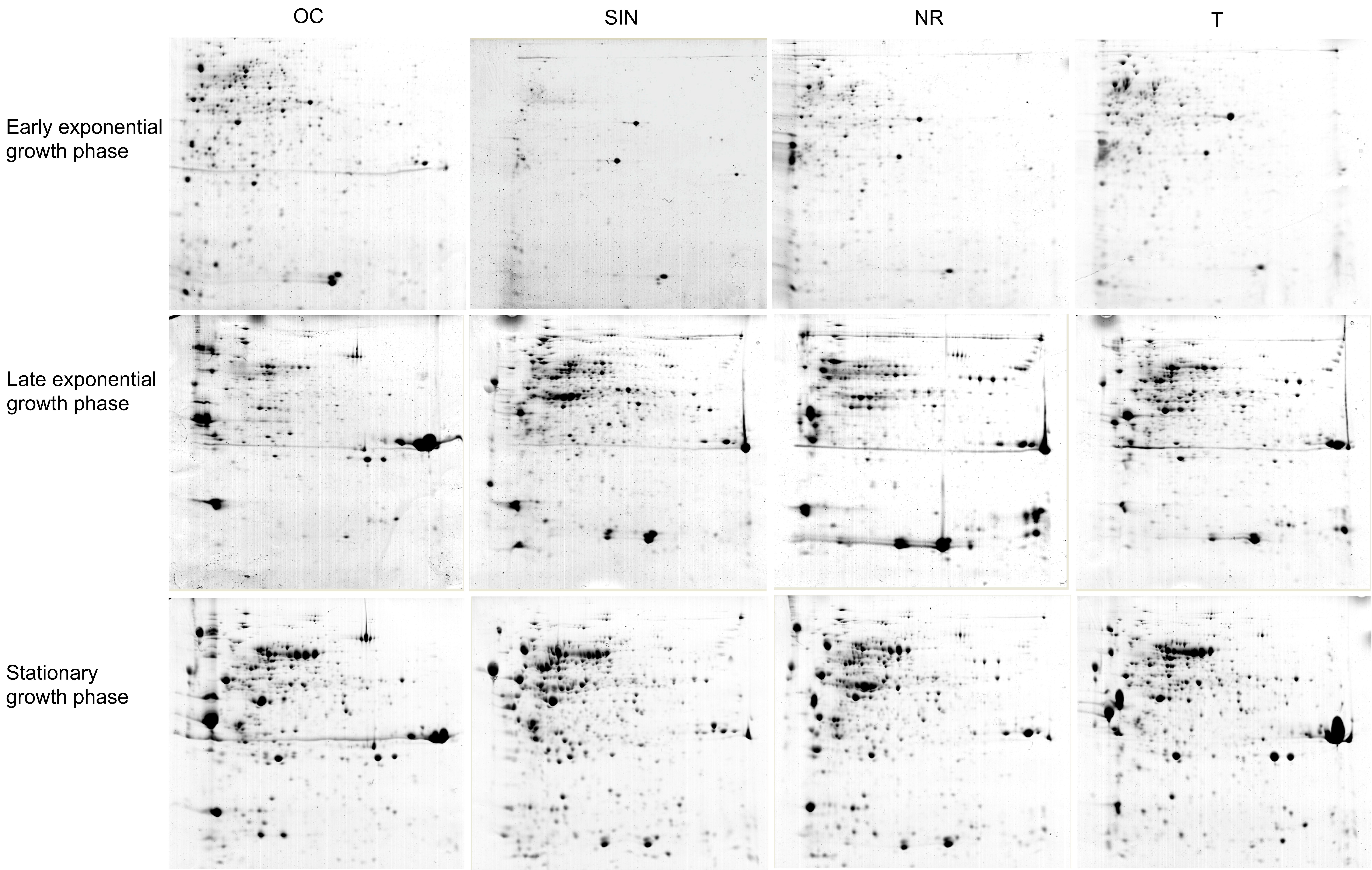
OC
SIN
NR
T
Early exponential growth phase
Late exponential growth phase
Stationary growth phase
