# Supplementary material for: Comparative secretome analysis of four isogenic Bacillus clausii probiotic strains
Source: Proteome Sci. 2013 Jul 1;11:28. doi: 10.1186/1477-5956-11-28 (PMC3716886; doi:10.1186/1477-5956-11-28)
Supplement: Additional file 6: Table S2 — Image analysis of 2-DE maps of secretome of the four B. clausii strains: Spots showing different mean intensity in the four maps are listed , together with the corresponding identified proteins. Reported values are the mean + SD of spot volumes (% vol) observed in the OC, SIN, NR, and T, proteomic electropherograms examined for each of three independent cultivation taken at the stationary (24 h) growth phase. Proteins are listed according to their respective match spot identification numbers (ID). The match ID is referred to those reported in Figure 4. [file 1477-5956-11-28-S6.docx]

**Tab. S. II**

**Tab. S. II. Image analysis of 2-DE maps of secretome of the four *B. clausii* strains:** Spots showing different mean intensity in the four maps are listed , together with the corresponding identified proteins. Reported values are the mean + SD of spot volumes (% vol) observed in the OC, SIN, NR, and T, proteomic electropherograms examined for each of three independent cultivation taken at the stationary (24h) growth phase. Proteins are listed according to their respective match spot identification numbers (ID). The match ID is referred to those reported in Fig. 4.

| **ID** | **Protein** | **OC** | **SIN** | **NR** | **T** |
| --- | --- | --- | --- | --- | --- |
| 33 | Oligopeptide ABC trasporters (ABC2414 and ABC 3090) | 3,542 ± 0,2745 | 0,783 ±0,1442 | 4,367 ± 0,1603 | 0,456 ± 0,1460 |
| 107 | Dihydrolipoamide dehydrogenase | 1,999 ± 0,2899 | 3,379 ± 0,1403 | 1,783 ± 0,1349 | 4,227 ± 0,2298 |
| 114 | Molecular chaperone GroEL | 0,127±0,0301 | 4,367±0,1148 | 3,857±0,0865 | 0,0207±0,00082 |
| 120 | Catalase | 3,339 ± 0,1992 | 1,475 ± 0,2405 | 1,028 ± 0,0283 | 1,620 ± 0,1814 |
| 156 | Catalase | 2,407 ± 0,2180 | 0,744 ± 0,2497 | 2,338± 0,2239 | 1,853 ± 0,1529 |
| 164 | Catalase | 2,243 ± 0,1311 | 2,198 ± 0,1136 | 4,510 ± 0,2396 | 2,618 ± 0,1915 |
| 207 | Dihydrolipoamide dehydrogenase,  Succinate semialdehyde dehydrogenase | 0,076 ± 0,0597 | 0,534 ± 0,2388 | 0,081 ± 0,0025 | 0,088 ± 0,0161 |
| 216 | Acetyl-CoA synthetase | 0,309±0,0036 | 2,768 ± 0,0431 | 0,868 ± 0,0633 | 0,041 ± 0,0168 |
| 231 | Glucose-6-phosphate isomerase | 0,364 ±0,1416 | 0,815 ± 0,1037 | 0,453 ± 0,0725 | 0,314 ± 0,1128 |
| 245 | Aminopeptidase | 0,508 ± 0,2273 | 2,078 ± 0,2640 | 0,570 ± 0,2258 | 0,328 ± 0,2296 |
| 247 | Mn 2+ / Zn 2+ ABC Transporter | 1,062 ±0,0278 | 7,793 ± 0,0868 | 1,072 ± 0,0135 | 1,148 ± 0,2697 |
| 281 | Trascriptional regulator | 0,299 ±0,2228 | 0,517 ± 0,1084 | 0,241 ± 0,1448 | 0,305 ± 0,3080 |
| 309 | Enolase | 2,180 ±0,2965 | 2,665 ± 0,1285 | 2,209 ± 0,1800 | 0,647 ± 0,1234 |
| 382 | Alcohol dehydrogenase | 0,085 ±0,0339 | 0,752 ± 0,0534 | 3,322 ± 0,0657 | 0,022 ± 0,0230 |
| 439 | Sugar phosphate isomerase,  Malate dehydrogenase | 4,253 ±0,2352 | 3,696 ± 0,1690 | 2,278 ± 0,0327 | 1,654 ± 0,0658 |
| 451 | Flagellin | 1,914 ± 0,2284 | 1,219 ± 0,2337 | 5,637 ± 0,1591 | 9,179 ± 0,2470 |
| 485 | Carbonic anydrase | 12,095 ± 0,2679 | 0,129 ± 0,0418 | 0,148 ± 0,2029 | 6,757 ± 0,2754 |
| 528 | Acethlmuramoyl-L-alanine amidase | 7,807 ± 0,4017 | 0,777 ± 0,1189 | 3,853 ± 0,1439 | 4,624 ± 0,2169 |
| 532 | Alkaline protease | 8,237 ± 0,2762 | 0,332 ± 0,1813 | 0,411 ± 0,1800 | 16,819 ± 0,2987 |
| 593 | Chain A Alkaline M- protease | 0,642 ± 0,0878 | 0,031 ± 0,0234 | 0,003 ± 0,0006 | 1,051 ± 0,2066 |
| 602 | Manganese superoxide dismutase | 2,638 ± 0,1245 | 1,378± 0,1755 | 1,079 ± 0,7244 | 0,0046 ± 0,0004 |
| 639 | Hypothetical protein ABC0920 | 4,746 ± 0,169 | 0,5786± 0,0079 | 6,283 ± 0,7244 | 1,223 ± 0,1760 |
| 659 | Hypothetical protein ABC2092 | 0,736 ± 0,112 | 0,367± 0,0079 | 0,391 ± 0,073 | 0,069 ± 0.012 |
| 673 | Hypothetical protein ABC2018 | 0,967 ± 0,1456 | 0,029±0,2270 | 0,088 ± 0,2268 | 0 |
| 674 | Two component response regulator | 0,006 ± 0,2745 | 1,594±0,1442 | 1,624 ± 0,1603 | 0,0028± 0,1460 |
| 694 | 2-Cys peroxiredoxine | 0,00345 ± 0,00121 | 0 | 1,787 ± 0,02983 | 0 |
